# Root colonization and growth promotion of soybean, wheat and Chinese cabbage by *Bacillus cereus* YL6

**DOI:** 10.1371/journal.pone.0200181

**Published:** 2018-11-21

**Authors:** Yongli Ku, Guoyi Xu, Xiaohong Tian, Huiqin Xie, Xiangna Yang, Cuiling Cao

**Affiliations:** 1 College of Life Sciences, Northwest A&F University, Yangling, P.R. China; 2 College of Resources and Environment, Northwest A&F University, Yangling, P.R. China; Henan Agricultural University, CHINA

## Abstract

Although phosphate-solubilizing bacteria (PSBs) are used in agricultural production, comprehensive research on PSB that colonize the rhizosphere of different plants and promote plant growth is lacking. This study was conducted to examine the growth-promoting effects and colonizing capacity of strain YL6, a PSB. YL6 not only increased the biomass of soybean and wheat in pot experiments but also increased the yield and growth of Chinese cabbage under field conditions. The observed growth promotion was related to the capacity of YL6 to dissolve inorganic and organic phosphorus and to produce indole-3-acetic (IAA) and gibberellin (GA). After applying YL6 to soybean, wheat and Chinese cabbage, the rhizosphere soil available phosphorus (available P) content increased by 120.16%, 62.47% and 7.21%, respectively, and the plant total phosphorus content increased by 198.60%, 6.20% and 78.89%, respectively, compared with plants not treated with YL6. To examine plant colonization, YL6 labeled with green fluorescent protein (YL6-GFP) was inoculated into the plant rhizosphere and found to first colonize the root surface and hairs and then to penetrate into the intercellular spaces and vessels. Collectively, these results demonstrate that YL6 promotes the growth of three different crops and colonizes them in a similar manner. The findings therefore provide a solid foundation for probing the mechanisms by which PSB affect plant growth.

## Introduction

As an essential element, phosphorus is the primary growth-limiting factor for plant and has significant functions in major metabolic processes, such as photosynthesis, respiration, and molecular synthesis [1]. In addition, phosphorus as a nonrenewable resource has attracted much attention [2]. In soil, the primary forms of phosphorus are apatite, calcium phosphate and organic phosphorus, the availabilities of which for plants are relatively low [3]. Moreover, deficiency in readily available P restricts crop yield. To address this problem, large amounts of phosphate fertilizers must be applied; however, 80–90% is fixed as insoluble P, and only a very small proportion is available to plants [4]. Furthermore, soil accumulation of insoluble P leads to environmental pollution [5]. Thus, approaches to increase the efficiency of phosphate fertilizers and to mitigate soil insoluble P pollution are urgently required.

Phosphate-solubilizing bacteria (PSBs) are required for a series of biochemical reactions to convert insoluble P to absorbable and available forms [6,7]. The primary mechanisms underlying these reactions include (a) secretion of organic acids, such as gluconic, acetic, and citric acids, to dissolve mineral complexes and (b) production of phosphatase enzymes to degrade insoluble organic P [8,9]. Similar to other plant growth-promoting rhizobacteria (PGPRs), some PSB strains can promote plant growth by producing indole-3-acetic (IAA) and gibberellin (GA) [10,11]. Therefore, application of PSBs in agricultural production is an important approach for achieving efficient phosphorus cycling and sustainable development in farmlands. To date, many studies have demonstrated that PSB application to the plant rhizosphere can improve plant growth [12]. For example, Walpola and Yoon [13] verified the promoting effects of PSBs on tomato growth. MK Abbasi [14] found that PSBs improve phosphorus deficiency in soil and plants, thereby promoting an increase in maize biomass. Additionally, M Tahir et al.[15] showed that the combined application of bio-organic phosphate and PSBs could result in a higher wheat yield with low fertilizer input under an arid climate, providing a new option for eco-friendly agricultural development.

Studying the colonization and distribution of endophytic bacteria in plants enriches our knowledge about how bacteria affect plant growth. Green fluorescent protein (GFP), first isolated from *Aequorea victoria*, is widely employed as a marker for studying gene expression and bacterial localization [16,17]. Furthermore, as GFP-labeled bacteria are easily detected without isolation, culture and identification, this is an ideal tool for studying plant colonization by endophytic bacteria. In fact, many PGPRs tagged with GFP have been utilized to examine microbial colonization patterns [18–20].

However, because of a lack of systematic studies, it remains unclear whether PSB strains can promote the growth of and colonize different crops in a similar way. In this study, strain YL6, a bacterium that dissolves inorganic and organic phosphorus, was applied to soybean (*Glycine max* L. Merr.), wheat (*Triticum aestivum* L.) and Chinese cabbage (*Brassica rapa* L., *Chinensis Group*) under different conditions. Additionally, YL6-GFP, GFP-tagged YL6, was employed to evaluate PSB colonization of the three different crops. Strain YL6 similarly promoted the growth of these plants by dissolving inorganic and organic phosphorus and producing IAA and GA in soil. Moreover, YL6-GFP was demonstrated to colonize these different plants in a similar manner.

## Materials and methods

### Ethics statement

This work was conducted in our scientific research field for PSB *Bacillus cereus* studies, which is owned by our institution. Therefore, no specific permission was required for using the locations or performing the study. This field did not involve endangered or protected species.

### Test strain

PSB strain YL6 maintained by our laboratory was isolated from the rhizosphere of Chinese cabbage at a soil depth of 10 cm at the Yangling Experiment Farm of Northwest A&F University (34.30° N, 108.08° E), Shaanxi, China. Based on 16s rRNA sequencing, YL6 was identified as *Bacillus cereus*. *The* GenBank accession number is KX580383 [9].

### Phosphate-solubilizing capacities and growth-promoting substances of YL6

Purified YL6 was inoculated into 100 ml of inorganic phosphorus liquid medium (glucose 10 g/L, (NH_4_)_2_SO_4_ 0.5 g/L, NaCl 0.3 g/L, KCl 0.3 g/L, MgSO_4_•7H_2_O 0.3 g/L, FeSO_4_•7H_2_O 0.03 g/L, MnSO_4_•4H_2_O 0.03 g/L, CaCO_3_ 10 g/L, pH 7) [21] and incubated in triplicate for 24, 48 and 72 h at 30°C with shaking at 180 rpm to measure soluble P and organic acids in the culture medium. For each repetition, 20 ml of the medium was centrifuged at 13,000×g for 10 min to obtain a cell-free supernatant. The soluble P content of the supernatant was determined using the molybdate blue method [22]. To evaluate organic acids by high-performance liquid chromatography (HPLC), the supernatant was filtered through 0.45-μm nylon filters (Millipore Corp, Billerica, MA, USA), and 20 μL was analyzed by HPLC (Essentia LC-15C, Japan) using a C-18 column, a flow rate of 1 mL/min and 90:10 (v/v) methanol-phosphate buffer (10 mmol/L; pH 2.7) as the mobile phase, with monitoring at 210 nm [23–25]. Additionally, purified YL6 was inoculated into 100 mL of organic phosphorus liquid medium (Extractum carnis 5 g/L, peptone 10 g/L, NaCl 5 g/L, pH 7.0; after 20 min, the medium was sterilized at 121°C, and 40 mL of a mixture of yolk and physiological saline at a ratio of 1:1 was added) [26] in triplicate and incubated at 30°C for 24 h with shaking at 180 rpm. The production of soluble P was measured using Molybdenum blue colorimetric method and acid, the alkalinity and the neutral phosphatase activity of YL6 were measured using the *p*-nitrophenyl phosphate method [27].

Purified YL6 cells were inoculated into 100 mL of LB liquid medium in triplicate and incubated at 30°C for 24 h with shaking at 180 rpm. For each repeat, 20 mL was centrifuged at 13,000×g for 10 min to obtain a cell-free supernatant, which was used to measure the production capacity of IAA using the Salkowski colorimetric assay [28] and of GA using fluorimetry assay [29].

### Influences of YL6 on rhizosphere soil and the biomass of different crops

#### Soybean pot experiment

Calcareous soil that had not been planted or fertilized for many was collected from the Medicinal Garden of Northwest A&F University (34.25° N, 108.06° E), air-dried and sifted through a 2-mm sieve. The bottom diameters and heights of the test pots were 28 cm. Each pot was filled with 3 kg of soil, and 6 soybean (Zhonghuang 13; previously purchased) seeds were sown per pot. YL6 strain was incubated at 30°C and 180 rpm for 48 h in LB liquid medium to produce a YL6 agent. The experiment involved three treatments: (1) YL6, 180 ml of 2.8×10^8^ cfu/ml YL6 agent added at soybean seedling emergence; (2) M, pure medium with no cells added; and (3) CK, the no-treatment control. All treatments were repeated five times. After germination, two seedlings in good growth condition were retained in each pot. The soil absolute water content was maintained at 20–22%. When three compound leaves had emerged, 8 seedlings were randomly collected for each of the treatments.

Root and shoot fresh and dry weights and the total fresh and dry biomass of soybeans were measured directly. Dry weights were determined after drying for 48 h at 80°C in an air oven; the R/S ratio was calculated the ratio of root dry weight and shoot dry weight [30]. Soybean vegetative growth and bean pod indices (including stem diameter, primary branch number, and pod length, width and number) were directly measured.

Fresh soil (2 g) was collected from the soybean rhizosphere was placed in a triangular bottle filled with 18 mL of sterile water and shaken at 30°C and 180 rpm for 30 min. This mixture was allowed to settle. Suspensions (1 mL) of the mixture were diluted to 10^−5^ and uniformly smeared on inorganic phosphorus agar medium using the plate-smearing method [31]. The plates were incubated (in darkness at 30°C) in a constant-temperature incubator for 3 days, after which the colony numbers of PSBs in the soil samples were assessed (cfu/g). Soil available P was extracted using the bicarbonate method and measured with the molybdate blue method [32]. Dried individual plants were ground, sifted through a 1-mm sieve and treated with H_2_SO_4_-HClO_4_; the phosphorus content was measured using the vanadium molybdenum yellow method [33].

#### Wheat pot experiment

In this experiment, we used wheat (Xiaoyan 22) seeds stored in our laboratory. The soil and pots were the same as those described above. Each pot was filled with 3 kg of soil, with 20 wheat seeds per pot. The 3 treatments were as follows: (1) YL6, 180 mL of 2.8×10^8^ cfu/mL YL6 agent added at seedling emergence; (2) M, pure medium with no strain added; and (3) CK, the no-treatment control. At the 3-leaf stage, 15 seedlings in good growth condition were retained in each pot. Each treatment was repeated 3 times. The soil absolute water content was maintained at 20–22%. At the tillering stage, 36 identically growing plants were randomly selected from 3 pots for each treatment.

The height, root length, root fresh and dry weights, shoot fresh and dry weights and total fresh and dry plant biomass of the different treatments were measured directly. The same methods as described above were used to determine soil available P, plant phosphorus and the number of phosphate-solubilizing rhizosphere bacteria.

#### Field experiment for Chinese cabbage

Seeds of Chinese cabbage (Shanghaiqing) were stored in our laboratory. The experiment was conducted at the Yangling Experiment Farm of Northwest A&F University (34.30° N, 108.08° E) from August 29 to October 10, 2016. The soil used was calcareous soil not cultivated with any crops for many years. The three treatments were as follows: (1) YL6, 1500 mL of 2.8×10^8^ cfu/mL YL6 agent added at cabbage seedling emergence; (2) M, pure medium with no strain added; and (3) CK, the no-treatment control. The experimental fields were divided into 15 identical plots (2 m×2 m), and the design was a randomized block with three replicates. The experimental farmland was managed using standard procedures during the Chinese cabbage growing season. Ripe vegetables in the same growth condition were randomly sampled for analysis. Root fresh and dry weights, shoot fresh and dry weights and total fresh and dry plant biomass of the different treatments were measured directly. To measure the quality of Chinese cabbage, the vitamin C content was determined using the molybdenum blue colorimetric method, cellulose and soluble sugar were evaluated using the anthrone colorimetric method, soluble protein was determined using the Coomassie blue staining method, and nitrate nitrogen content was assessed by nitration of salicylic acid colorimetry [34].

### YL6 colonization on root surfaces of soybean, wheat and Chinese cabbage seedlings

GFP-labeled YL6 was constructed in our laboratory, and stability was tested [9]. Soybean, wheat and Chinese cabbage were cultured using the sand culture method. To this end, sand (passed through a 24-mesh sieve) was washed clean with tap water and sterilized at 120°C in an oven for 48 h. Next, 1 kg of sterilized sand was added to individual boxes (195 mm×146 mm×65 mm), followed by tap water to maintain the absolute water content range within 8–12%. Soybean, wheat and Chinese cabbage seeds were sterilized with 10 mL of 2% NaClO (13% active Cl^-^ content) for 10 min and rinsed 5 times with sterile water. The sterile seeds were soaked in warm water for 30 min, and those that floated on the surface of the water were removed. The remaining seeds were evenly sown on three sheets of wet, stacked filter paper in culture dishes. The culture dishes were incubated at 28°C in the dark in an incubator for 2 days. Water was added every 8 h to keep the filter papers wet. After germination, seeds were incubated at 28°C under a 16/8-h photoperiod [35]. The seeds were transplanted into different culture boxes with sand. When 3 primary leaves had emerged and a root system had developed, the YL6-GFP bacterial suspension (2.8×10^8^ cfu/mL) diluted twice (1.35×10^8^ cfu/mL) was added to the culture boxes. Seedlings were collected after 3, 6 and 9 days and rinsed with sterile water. For bacterial colony observations, root tissues were cut into tiny pieces in a crisscrossed pattern using a sterilized razor blade, and the pieces were placed in glass dishes using the hydrostatic tablet compressing method. YL6-GFP colonization on fresh roots was visualized by fluorescence microscopy (Olympus CCD-DP26).

### Statistical analyses

Microsoft Excel 2007 was employed to process the data. Comparisons among treatments were performed using the least significant difference (LSD) approach, with significance at *P*<0.05, in SPSS 23.0.

## Results

### Phosphate-solubilizing capacity and production of growth-promoting substances of YL6

Due to the differences in the mechanisms by which YL6 dissolves inorganic and organic phosphorus, the phosphorus-solubilizing capacities of strain YL6 in different liquid culture media were investigated (Table 1). First, YL6 was inoculated into organic phosphorus liquid medium to determine acid phosphatase, alkaline phosphatase and neutral phosphatase activities and the accumulation of available P at various time points. The activities of these phosphatases were highest when the content of available P in the medium was greatest, with the concentration of available P increasing to 152.253 μg/ml after 48 h of culture. Additionally, YL6 secreted oxalic, malonic and succinic acids to increase available P in the inorganic phosphorus liquid medium (Table 1). Other growth-promoting substances were also detected. For example, the concentrations of IAA and GA increased to 2.581 mg/L and 1.425 mg/L, respectively, after 24 h of incubation (Table 1). These results indicate that strain YL6 had strong capacities to dissolve different forms of phosphorus into P available to plants.

**Table 1:** The ability of YL6 to dissolve phosphorus and produce hormones.

Time	Organic phosphorus liquid medium inoculated with YL6				Inorganic phosphorus liquid medium inoculated with YL6				LB liquid medium inoculated with YL6	
	Alkaline phosphatase	Acid phosphatase	Neutral phosphatase	Soluble P	Oxalate	Malonic acid	Succinic acid	Soluble P	IAA	GA
	μg pNP/ mL/ h	μg pNP / mL/ h	μg pNP/ mL/ h	mg/ mL	μg/ mL	mg / mL	mg/ mL	μg/ mL	mg/ L	mg/ L
**24 h**	**25.913±0.759b**	**27.863±0.006b**	**25.433±0.056b**	**128.635±1.767c**	**1085.95±90.71b**	**1011.47±22.64b**	**405.71± 6.83b**	**5.122±0.531b**	**2.581±0.270c**	**1.425±0.015a**
**48 h**	**28.993±0.719a**	**31.140±0.231a**	**30.942±0.360a**	**162.114±3.635a**	**1306.15±11.90a**	**1058.20±48.63a**	**593.71±2.95a**	**9.329±0.214a**	**4.843±0.110a-**	**1.173±0.001b**
**72 h**	**26.309±0.040b**	**25.188±0.752b**	**26.789±0.719b**	**152.253±3.437b**	**1042.41±30.93b**	**621.84±70.04c**	**373.55±3.17c**	**8.721±0.991a**	**3.801±0.133b-**	**1.171±0.013b-**

Values are the mean±SE of three replicates. Within each column, values followed by the same letter are not significantly different, according to Fisher's protected LSD test (*P* < 0.05).

### Influence of YL6 on the biomass and quality of the different crops

**Fig 1:**
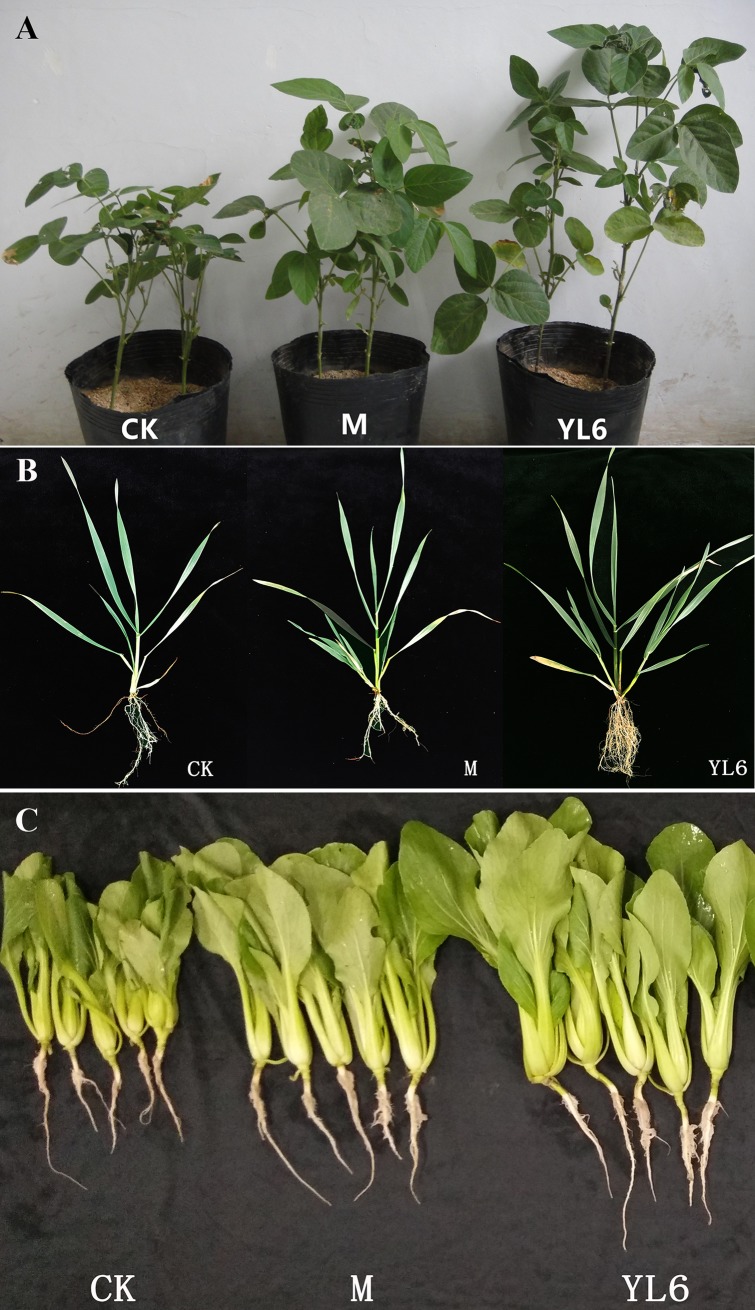
Effect of YL6 on growth promotion in soybean, wheat, and Chinese cabbage. **CK**, control group; **M**, treatment with liquid medium without the strain; **YL6**, treatment with the YL6 agent. **A**. Soybean, **B**. wheat, **C**. Chinese cabbage.

To test whether this PSB is able to promote plant growth, soybean, wheat and Chinese cabbage carrying were planted and treated with YL6. Based on the phenotypes of these crops, YL6 treatment notably improved growth (Fig 1A–1C). Furthermore, the fresh and dry weights of roots, shoots and whole plants treated with strain YL6 were much higher than those of the M and CK groups (Table 2). For soybean, the R/S ratio of the YL6 group increased by 28.8% (*P* < 0.05) compared with that of the CK group. Different indices of soybean growth and bean pods (stem diameter, primary branch number, and pod length, width and number) also increased significantly with YL6 inoculation compared with the control (Table 3), and the number, length and width of pods increased significantly by 16.81%, 94.34% and 251.13%, respectively (*P* < 0.05). Compared with the CK and M groups, YL6 increased wheat and Chinese cabbage biomass. In addition to biomass, the nutritional value of Chinese cabbage was also determined, and we found that the leaf vitamin C, soluble sugar, soluble protein and cellulose contents were highest with YL6 treatment, reaching 2.105, 15.610, 15.695 and 42.539 mg/g (*P*<0.05), respectively (Table 4). Another important index of Chinese cabbage is the content of nitrate nitrogen, which can be transformed into nitrates harmful to humans. Although the nitrate nitrogen content did not differ significantly among the groups (*P*<0.05), it was lower in the YL6 group (Table 4). Based on these results, the PSB YL6 significantly improved the growth of these crops.

**Table 2:** Effect of YL6 on the biomass of soybean, wheat and Chinese cabbage.

Crop type	Treatment	Shoot fresh weight	Root fresh weight	Shoot dry weight	Root dry weight	Fresh biomass	Dry biomass	R/S (DW/DW)	Yield/plot
		g/plant^-1^	g/plant^-1^	g/plant^-1^	g/plant^-1^	g/plant^-1^	g/plant		kg
**Soybean**	**CK**	**2.72±0.06c**	**1.36±0.06c**	**0.42±0.02c**	**0.10±0.04c**	**4.08±0.15c**	**0.52±0.01c**	**0.25±0.02b**	**-**
	**M**	**3.47±0.19b**	**1.52±0.05b**	**0.61±0.00b**	**0.20±0.01b**	**4.99±0.057b**	**0.81±0.01b**	**0.32±0.01a**	**-**
	**YL6**	**4.65±0.10a**	**2.75±0.07a**	**0.74±0.02a**	**0.24±0.01a**	**7.40±0.14a**	**0.97±0.02a**	**0.32±0.01a**	**-**
**Wheat**	**CK**	**0.55±0.18c**	**0.04±0.00b**	**0.14±0.02c**	**0.02±0.01c**	**0.65±0.26c**	**0.16±0.03c**	**0.16±0.01a**	**-**
	**M**	**1.13±0.16b**	**0.09±0.01b**	**0.27±0.01b**	**0.04±0.01b**	**1.15±0.04b**	**0.30±0.02b**	**0.13±0.01b**	**-**
	**YL6**	**2.32±0.20a**	**0.25±0.07a**	**0.57±0.00a**	**0.09±0.00a**	**2.66±0.15a**	**0.67±0.00a**	**0.16±0.00a**	**-**
**Chinese cabbage**	**CK**	**12.68±0.63c**	**0.47±0.06c**	**0.99±0.06c**	**0.08±0.02c**	**13.15±0.64c**	**1.07±0.07b**	**0.08±0.00b**	**2.21±0.09b**
	**M**	**22.93±1.01b**	**1.34±0.03b**	**1.66±0.18b**	**0.19±0.02b**	**24.07±0.96b**	**1.11±1.03b**	**0.11±0.02a**	**2.57±0.03b**
	**YL6**	**43.61±5.62a**	**1.57±0.23a**	**2.74±0.29a**	**0.25±0.05a**	**44.76±5.33a**	**3.04±0.24a**	**0.11±0.02a**	**3.87±0.39a**

Values are the mean±SE of three replicates. Within each column, the values of the same crops followed by the same letter are not significantly different, according to Fisher's protected LSD test (*P* < 0.05). Note: R/S is the abbreviation of root-shoot ratio, which refers to the ratio of the dry weight of root to the shoot part.

**Table 3:** Effect of phosphate-solubilizing bacteria on soybean vegetative growth and pod indices.

Treatment	Stem diameter	Primary branch	Pod length	Pod width	Pod
	(cm)	number	(cm)	(cm)	number
**CK**	**0.39±0.018b**	**4.67±0.577b**	**3.45±0.071b**	**0.53±0.058b**	**1.33±0.577b**
**M**	**0.41±0.031b**	**6.33±1.155b**	**3.50±0.000b**	**0.53±0.058b**	**1.33±0.577b**
**YL6**	**0.51±0.026a**	**11.00±2.000a**	**4.03±0.058a**	**1.03±0.058a**	**4.67±0.577a**

Values are the mean±SE of three replicates. Within each column, values followed by the same letter are not significantly different,according to Fisher's protected LSD test (*P* < 0.05).

**Table 4:** Effect of different treatments on the quality of Chinese cabbage.

Treatment	Cellulose	Vitamin C	Soluble sugar of leaves	Soluble protein of leaves	Nitrate nitrogen of leaves mg/g
	mg/g	mg/g	mg/g	mg/g	
**CK**	**6.445±0.337b**	**1.986±0.079a**	**12.004±1.246b**	**19.817±0.469c**	**1.140±0.116a**
**M**	**7.575±0.569b**	**2.059±0.140a**	**13.305±0.831b**	**31.108±1.713b**	**1.064±0.069a**
**YL6**	**15.610±2.613a**	**2.105±0.127a**	**15.695±0.672a**	**42.539±4.491a**	**1.062±0.052a**

Values are the mean±SE of three replicates. Within each column, values followed by the same letter are not significantly different, according to Fisher's protected LSD test (*P* < 0.05).

### Plant phosphorus, number of soil phosphate-solubilizing bacteria and soil available P

The amount of plant phosphorus was assessed to determine whether strain YL6 promotes soybean, wheat and Chinese cabbage growth by facilitating soil phosphorus uptake. First, PSBs in the rhizosphere soil of these crops were counted, revealing an increase in PSB abundance with the application of YL6 to 13-, 35- and 10-fold those of the CK group in soybean, wheat and Chinese cabbage, respectively (Table 5). The increase in PSBs generated more available P in the soil (Table 5). For example, the soil available P content in the YL6 group was 120.16% higher than that in the CK group in the soybean pot experiment. Moreover, the increased amount of available soil P was readily utilized by the plants, which led to an increase in the total P content in soybean, wheat and Chinese cabbage (Table 5). The total P content of soybean, wheat and Chinese cabbage in the YL6 group was 198.60, 6.20 and 78.89% higher than those in the CK groups, respectively. These results indicate that strain YL6 similarly promoted the growth of different crops by increasing available soil P.

**Table 5:** Effect of YL6 on the number of phosphate-solubilizing bacteria in rhizosphere soil, the soil available phosphorus content and the plant phosphorus content.

Crop	Treatments	PSB number in rhizosphere soil 1×10^5^ cfu/g	Soil available phosphorus mg/kg	Plant phosphorus mg/g
**Soybean**	**CK**	**0.009c**	**2.530±0.050b**	**1.220±0.050c**
	**M**	**0.08b**	**2.720±0.030b**	**2.081±0.030b**
	**YL6**	**0.12a**	**5.570±0.230a**	**3.643±0.230a**
**Wheat**	**CK**	**0.01c**	**13.788±0.654c**	**1.579±0.016b**
	**M**	**0.16b**	**18.942±1.332b**	**1.671±0.030a**
	**YL6**	**0.35a**	**22.401±1.378a**	**1.677±0.032a**
**Chinese cabbage**	**CK**	**0.35c**	**18.898±1.976b**	**1.179±0.212b**
	**M**	**1.80b**	**19.254±1.793b**	**1.395±0.000b**
	**YL6**	**3.50a**	**20.261±1.744a**	**2.109±0.130a**

Values are the mean±SE of three replicates. Within each column, the values of the same crops followed by the same letter are not significantly different, according to Fisher's protected LSD test (*P* < 0.05).

### Root colonization of the different crops by YL6

**Fig 2:**
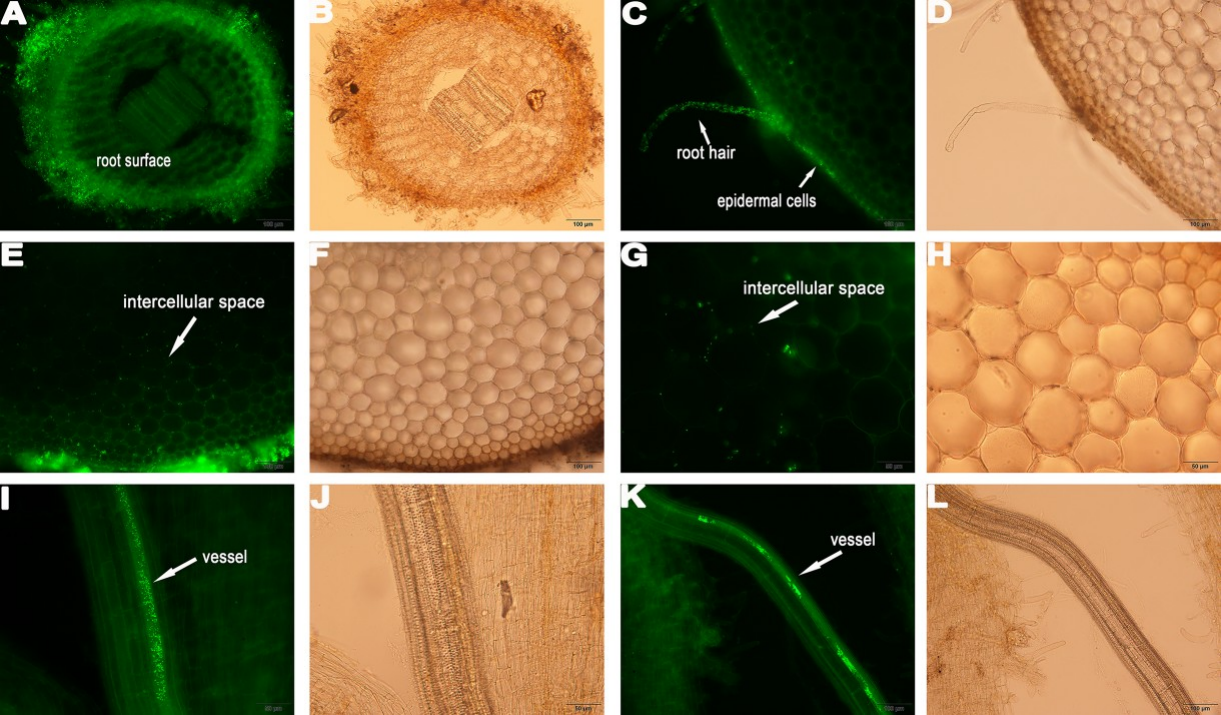
Colonization process of YL6-GFP in soybean root tissue. **A** longitudinal picture of the root; **C** picture of root hairs and the primary root surface; **E**, **G** intercellular space of the cortex; **I**, **K** YL6-GFP in the vessels of branch roots. **B**, **D**, **F**, **H**, **J**, and **L** were obtained under bright-field microscopy.

**Fig 3:**
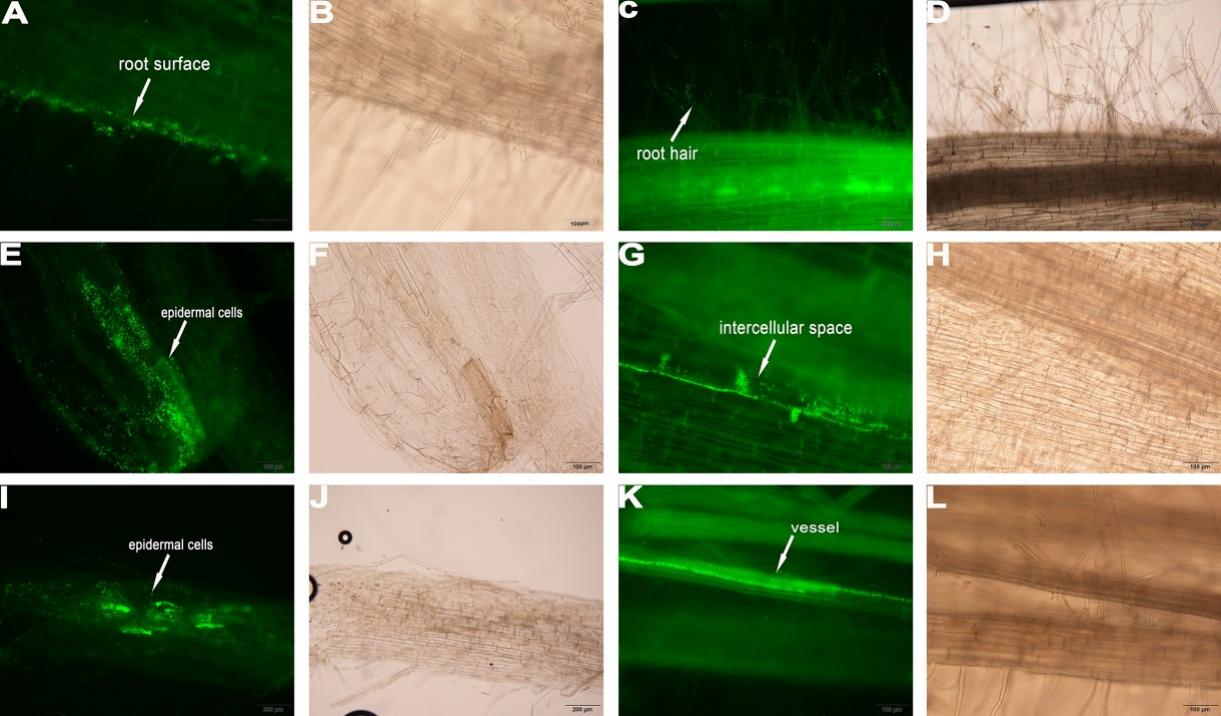
Colonization process of YL6-GFP in wheat root tissue. **A**, **C** surface of the primary root and root hairs; **E**, **I** some epidermal cells colonized by YL6-GFP; **G** some bacteria distributed in the intercellular spaces between cells; **K** YL6-GFP in the vessels of branch roots. **B**, **D**, **F**, **H**, **J**, and **L** were obtained under bright-field microscopy.

**Fig 4:**
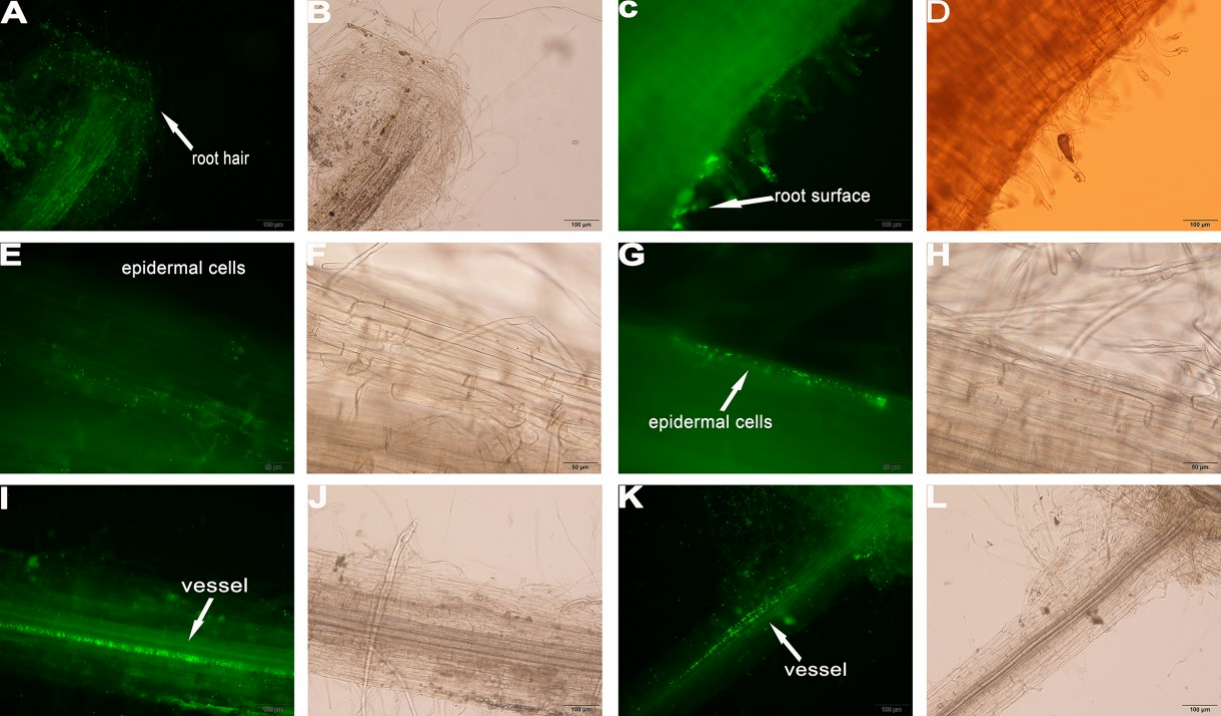
Colonization process of YL6-GFP in Chinese cabbage root tissue. **A**, **C** YL6-GFP on the root hairs and surface of the primary root; **E**, **G** some epidermal cells colonized by YL6-GFP; **I**, **K** YL6-GFP in vessels. **B**, **D**, **F**, **H**, **J**, and **L** were obtained under bright-field microscopy.

In addition to evaluating the capacities of the PSB to promote crop growth, colonization by a GFP-tagged strain of PSB, YL6-GFP, was examined. Under fluorescence microscopy, YL6-GFP was detected only at the root hairs of the three crops at three days after inoculation, suggesting that YL6-GFP first attached to the root hair surfaces and then penetrated into the root hairs (Fig 2A and 2C; Fig 3A and 3C; Fig 4A and 4C). In soybean, many fluorescent foci were observed in the intercellular spaces of the root cortex on the sixth day after inoculation (Fig 2E and 2G). YL6-GFP was also distributed in the intercellular spaces of and even inside epidermal cells (Fig 3E, 3G and 3I). Additionally, the strain colonized the surfaces of Chinese cabbage epidermal cells (Fig 4G). In addition, YL6-GFP had entered the vessels of these plants because green fluorescence was detected in the vessels of branch roots, roots and different samples of soybean, wheat and Chinese cabbage on the ninth day after inoculation (Fig 2I and 2K; Fig 3K; Fig 4I and 4K). These results suggest that YL6-GFP colonized these different plants through a similar process: first attaching to the root hair, penetrating into the vessels and finally expanding into other organs.

## Discussion

In this study, the effects of a PSB on three common crops, soybean, wheat and Chinese cabbage, were examined. Soybean is one of the most important crop plants grown for seed proteins and vegetable oil [36], and wheat is widely planted worldwide, as the caryopsis is one of the staple foods for humans. Chinese cabbage is a dicotyledonous plant that is a popular leaf vegetable in China [37].

In this study, the capacities of *Bacillus cereus* strain YL6 to dissolve inorganic and organic P were examined. In general, PSBs solubilize insoluble P by secreting organic acids or enzymes [38]. According to our results, YL6 not only produced oxalic, malonic and succinic acids but also secreted acidic, neutral, and alkaline phosphatases. The contents of these organic acids were highest at 48 h, and similarly, the activities of the three types of phosphatases peaked at 48 h. This result indicates that 48 h is the optimum time frame for YL6 to dissolve insoluble P under laboratory conditions. The content of soluble P increased rapidly in the first 48 h, which was most likely because the many acids produced and phosphatases secreted promoted PSB transformation of insoluble P into soluble P [39] [6]. Increased P availability enhances the root growth of plants and the yield of crops. For example, Sharon isolated one efficient PSB that increased tomato growth [40]. Furthermore, crop growth was promoted by YL6 IAA and GA secretion, a result supported by other publications [41–43]. These findings indicate that YL6 may promote plant growth by secreting IAA and GA.

Pot experiments with soybean and wheat were conducted to determine the influences of YL6 on the growth of these plants. The numbers of PSBs in the rhizosphere soil of both crops increased after the addition of YL6. Therefore, this PSB successfully penetrated into the soil and increased the amount of available soil P for crop absorption. The plant total P content, biomass and bean pod indices of soybean treated with YL6 were markedly higher than those in the other treatments. In the wheat pot experiment, plant biomass with YL6 addition was also notably higher than those of the groups not treated with YL6. However, the content of plant total P in the YL6 treatment was not significantly different between the medium treatments (*P*<0.05). Because of the higher biomass, the plants in the YL6 treatment utilized more available P than did the plants in the M treatment, suggesting that YL6 can enhance the available P utilization ratio, similar to Zhang’s report [44].

Based on the positive results obtained in the pot experiments, we also determined the effects of YL6 on the biomass and quality of Chinese cabbage under field conditions. The number of soil PSBs in the YL6 treatment was significantly higher than that in the other two groups, which demonstrated that YL6 can survive in soil under field conditions. The survival and colonization capacity of YL6 when inoculated in soil are prerequisites for this PSB to play an important role in the environment and are key for its phosphate-solubilizing and plant growth-promoting capacities. Greater abundance of these bacteria in the soil favors plant growth [45], and strain YL6 enhanced the growth of Chinese cabbage in the field.

PSBs primarily rely on their ability to transform insoluble P into available P [46–48]. YL6 inoculation increased soil available P, and Chinese cabbage directly absorbed and utilized this available P in soil to promote plant growth and total P accumulation. Therefore, YL6 inoculation markedly improved growth parameters such as root and shoot dry biomass, yield and total P uptake in Chinese cabbage compared with those of the control [46,49,50]. This result is consistent with the conclusions of Sundara, Akbari and Swarnalakshmi [49,51,52]. YL6 also improved nutritional quality indices of Chinese cabbage, such as soluble sugar, soluble protein, and in particular vitamin C. Vitamin C is a highly active substance that can promote immunity and prevent cancer, heart disease and stroke in humans but can also be employed for resisting the effects of aging and stress [53]. Soluble sugar is the material basis of polysaccharides, proteins, fats and other macromolecular compounds in plants, and our results for YL6 are consistent with those of Hui [54]. Nitrate nitrogen is also an important indicator of vegetable quality: because nitrite is carcinogenic and causes severe damage, improving vegetable quality through nitrate nitrogen reduction is important [55]. And in this study, the content of nitrate nitrogen of YL6 treatment was lowest among three treatments. These results demonstrated positive effects of YL6 that improved the quality of Chinese cabbage.

The survival and colonization of PSBs in the plant rhizosphere is the basis for plant growth promotion [56]. However, in many cases, PSBs do not achieve the desired effect because of insufficient numbers in the rhizosphere or failure of rhizosphere or plant colonization [57]. In this study, both pot and field experiments showed that YL6 universally promoted the growth of crops, and analysis of the colonization of crops by YL6 is critical. GFP-labeled YL6 was used to inoculate the rhizosphere of soybean, maize and Chinese cabbage seedlings, and fluorescence microscopy revealed that GFP-labeled YL6 colonized the root hairs, epidermal cells, cortex cells, intercellular spaces and vessels of soybean, wheat and Chinese cabbage seedling roots. The results of this study are consistent with those by other researchers [18,58–60]. Root hairs, root surfaces and epidermal cells are most likely primarily colonized by YL6 because of chemotaxis toward root exudates [61], as various carbohydrates, amino acids, organic acids and other compounds in plant root exudates are a source of nutrients for root-associated bacteria [62]. Additionally, YL6 may overcome cortical barriers by secreting cell wall-degrading enzymes (CWDEs) [63], allowing YL6 to colonize the nutrient-rich intercellular spaces of plant [64] and spread throughout the host through the lumen of xylem vessels [65].

## Conclusions

The experiments described above showed that YL6 not only dissolved soil insoluble P by secreting organic acids and phosphatases but also successfully colonized crop root tissues and promoted crop growth by secreting IAA and GA. YL6 inoculation promoted plant growth and quality and improved soil fertility. In conclusion, application of YL6 is a good cost-effective and environmentally friendly choice to achieve high yields and reduce chemical P fertilizer use. Further research on the long-term survival of PSBs under field conditions and PSB colonization mechanisms is required in the future.
