# Correction: Root colonization and growth promotion of soybean, wheat and Chinese cabbage by *Bacillus cereus* YL6

**DOI:** 10.1371/journal.pone.0210035

**Published:** 2018-12-27

**Authors:** Yongli Ku, Guoyi Xu, Xiaohong Tian, Huiqin Xie, Xiangna Yang, Cuiling Cao, Yinglong Chen

Dr. Yinglong Chen is not included in the author byline. He should be listed as the seventh author and affiliated with State Key Laboratory of Soil Erosion and Dryland Farming on the Loess Plateau, Northwest A&F University, Chinese Academy of Sciences, Yangling, Shaanxi 712100, China, and the UWA Institute of Agriculture, & School of Agriculture and Environment, The University of Western Australia, LB 5005, Perth 6001, Australia. The contributions of this author are as follows: Contributed resources, supervision, and validation.

There are errors in the Funding section. The correct funding information is as follows: This work was supported by National Natural Science Foundation of China (31471946) and Chinese Academy of Sciences (“100 Talent” Program, A315021449).
